# Effectiveness of eight or more antenatal contacts on health facility delivery and early postnatal care in low- and middle-income countries: a propensity score matching

**DOI:** 10.3389/fmed.2023.1107008

**Published:** 2023-07-21

**Authors:** Dagmawi Chilot, Fantu Mamo Aragaw, Daniel Gashaneh Belay, Melaku Hunie Asratie, Mehari Woldemariam Merid, Anteneh Ayelign Kibret, Nahom Worku Teshager, Adugnaw Zeleke Alem

**Affiliations:** ^1^Department of Human Physiology, College of Medicine and Health Science, School of Medicine, University of Gondar, Gondar, Ethiopia; ^2^Department of Epidemiology and Biostatistics, College of Medicine and Health Science, Institute of Public Health, University of Gondar, Gondar, Ethiopia; ^3^Department of Human Anatomy, College of Medicine and Health Science, School of Medicine, University of Gondar, Gondar, Ethiopia; ^4^Department of Women and Family Health, College of Medicine and Health Science, School of Midwifery, University of Gondar, Gondar, Ethiopia; ^5^Department of Pediatrics and Child Health, College of Medicine and Health Science, School of Medicine, University of Gondar, Gondar, Ethiopia

**Keywords:** antenatal care, health facility delivery, early PNC, propensity score matching, low- and middle-income countries

## Abstract

**Background:**

Despite progress in reducing maternal and child mortality, many low- and middle-income countries (LMICs) still experience an unacceptably high level of the problem. The World Health Organization (WHO) recently recommended pregnant women should have at least eight antenatal care visits (ANC8+) with a trained healthcare provider as a key strategy to promote pregnant women's health. Antenatal care is an imperative factor for subsequent maternal healthcare utilization such as health facility delivery and early postnatal care (EPNC). This study aimed to examine the net impact of ANC8+ visits on health facility delivery and EPNC in LMICs using a propensity score matching analysis.

**Methods:**

We used the recent Demographic and Health Survey (DHS) datasets from 19 LMICs. Women of reproductive age (15–49 years) who had given birth within 1 year preceding the survey were included. A propensity score matching analysis was employed to assess the net impact of eight or more antenatal care visits on health facility delivery and early postnatal care.

**Result:**

After matching the covariates, women who attended ANC8+ visits had a 14% (ATT = 0.14) higher chance of having their delivery at health facilities compared with women who attended less than eight ANC visits. This study further revealed that women who had ANC8+ visits were associated with a 10% (ATT = 0.10) higher probability of early PNC compared with their counterparts.

**Conclusion and recommendation:**

This study confirmed that ANC8+ visits significantly increased the likelihood of health facility-based delivery and early PNC utilization in LMICs. These findings call for public health programs to focus on pregnant women attending adequate ANC visits (according to revised WHO recommendation) as our study indicates that ANC8+ visits significantly improved the chances of subsequent care.

## Background

Despite progress in reducing maternal and child mortality, many low- and middle-income countries (LMICs) still experience an unacceptably high level of the problem (1, 2). In 2017, 295,000 women died due to complications in pregnancy and childbirth globally. The vast majority of these deaths (86%) occurred in Sub-Saharan Africa and Southern Asia (3). All countries with the United Nations have agreed and adopted 17 Sustainable Development Goals so that they can change the world for the better (4, 5). Sustainable development goal 3 (SDG-3) aims to reduce the global maternal mortality ratio to 70 deaths per 100,000 live births by 2030 (6). To achieve this goal, timely and frequent care for pregnant women by healthcare providers has paramount significance (7, 8).

The World Health Organization (WHO) recently recommended pregnant women should have at least eight antenatal care (ANC8+) visits with a trained healthcare provider as a key strategy to promote pregnant women's health (9, 10). Timely and frequent ANC endorses existing disease treatments, vaccination, malaria prophylaxis, iron supplementation, nutrition counseling, HIV counseling and testing, urinary tract infection treatment, and other related services (11–13). Antenatal care is also an imperative factor for subsequent maternal healthcare utilization such as health facility delivery and early postnatal care (EPNC) (14, 15). As advocated by the WHO, health facility delivery with trained birth attendants and EPNC have principal importance in reducing maternal mortality (16–18).

The first ANC visit should start in the first trimester while postnatal care should be given to a woman within 48 h after the delivery of the placenta and continue for 42 days (19, 20). In LMICs, numerous studies have revealed empirical evidence concerning the impact of individual, household, and community-level factors on this continuum of care utilization in different settings (21–24). However, the relationship between ANC and the subsequent continuum of care in a cross-sectional study might not be robust because of the presence of selection bias and confounding (25). Despite country-specific studies that have tried to link the ANC4+ visits with institutional delivery and early PNC (14, 25, 26), there is no evidence of the net impact of ANC8+ visits on subsequent institutional delivery and early PNC in LMICs. Many women in developing countries do not adhere to the WHO recommendation of at least eight ANC visits during pregnancy (27). Subsequent care aspects such as having delivery at a health facility and attending early postnatal care also remain a significant problem in those nations. Exploring the impact of ANC8+ on subsequent care can help to inform better policy and practice (28, 29). Therefore, our study aimed to examine the net impact of ANC8+ visits on health facility delivery and EPNC in LMICs using PSM and employing sensitivity analyses to assure the robustness of our findings.

## Materials and methods

### Study design and setting

This study used secondary data from 19 LMICs that have a recent Demographic Health Survey (DHS) collected after the 2016 WHO ANC model (30). This model recommends a minimum of eight ANC contacts during pregnancy, with the first contact occurring in the first trimester of gestation followed by two and five contacts in the second and third trimesters, respectively (9).

**Figure 1:**
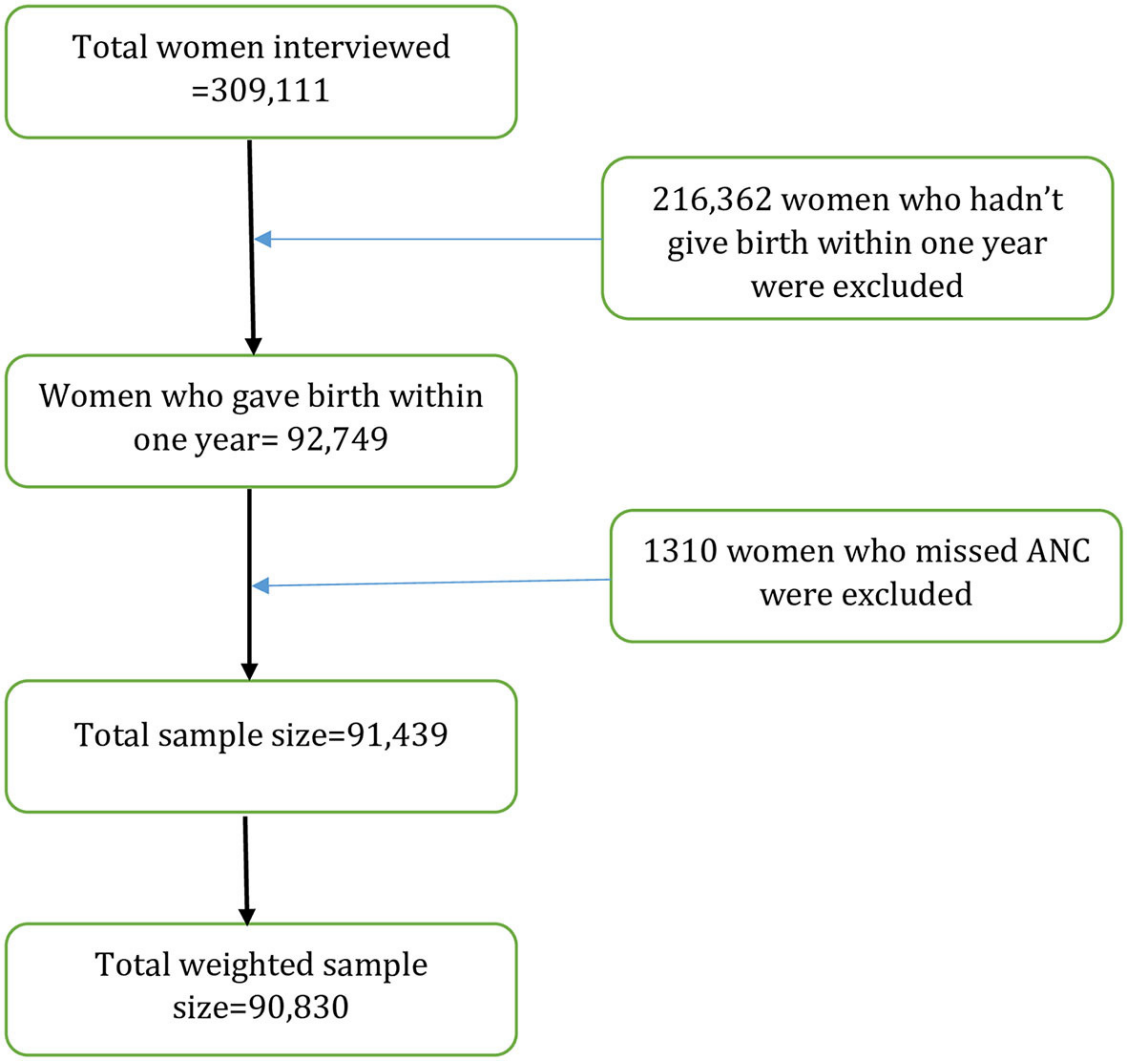
Diagrammatic representation of sample selection in the study.

The DHS is a nationally representative survey that uses a cross-sectional design to collect data on women, men, and children. The surveys use the same standardized data collection procedures, sampling, questionnaires, and coding, making the results comparable across countries. A total of 309,111 reproductive ages (15–49 years) were interviewed in 19 LMICs. However, this study was limited to women aged 15–49 years who had live births within the year prior to the surveys. Finally, 90,830 women aged 15–49 years were included in the analysis (Figure 1). The list of those countries and the respective year of surveys is provided in Table 1.

**Table 1:** Study setting and year of surveys.

**Country**	**Year of survey**	**Region**
Albania	2017/18	Southeastern Europe
Bangladesh	2017/18	South Asia
Benin	2017/18	West Africa
Cameroon	2018	Central Africa
Gambia	2019/20	West Africa
Guinea	2018	West Africa
India	2021	South Asia
Jordan	2017/18	Western Asia
Liberia	2019/20	West Africa
Madagascar	2021	East Africa
Mauritania	2020/21	Northwest Africa
Mali	2018	West Africa
Nigeria	2018	West Africa
Pakistan	2017/18	South Asia
Philippines	2017	Southeast Asia
Rwanda	2019/20	East Africa
Sierra Leone	2019	West Africa
Tajikistan	2017	Central Asia
Zambia	2018	Southern Africa

### Definition of variables

#### Outcome variable

The outcome variables of this study were health facility delivery and EPNC. Health facility delivery was a dichotomous variable where respondents were coded as having an institutional delivery if they delivered within a public, private, or non-governmental organization facility. Early PNC is a postnatal checkup from a skilled health provider within 2 days after childbirth. It was coded into a binary variable where women who had a postnatal checkup by a skilled provider within 2 days of delivery were coded 1 and otherwise 0.

#### Treatment variable

The treatment variable was an ANC visit of their most recent pregnancy that ended with a live birth. The number of ANC contacts of pregnant women was categorized as those who had at least eight ANC contacts and those who had fewer than eight ANC contacts, according to the 2016 WHO recommendation.

#### Matching variables

This study considered covariates on the basis of available literature that was related to both the treatment (ANC visits) and the outcome variables (health facility delivery and early PNC) (14, 25, 26). The lists of included variables were as follows: age, education level, household wealth status, marital status, wanted pregnancy, media exposure, birth order, residence, sex of household head, and accessing healthcare.

### Statistical analyses

This study used PSM to draw causal inferences about the effect of eight or more ANC on health facility delivery, and early PNC. PSM is a statistical method that allows us to evaluate the treatment effects for observational data, in cases where randomized controlled trials are either infeasible, unethical, or when researchers need to evaluate treatment effects from survey data (31). It reduces bias due to observable individual characteristics by matching and helps create comparable balanced groups of respondents with respect to observed covariates (25).

STATA V.14.2 software was used to clean, recode, and analyze the data. Pscore Stata command was used to generate the propensity score including covariates associated with ANC visits (treatment variable) and both outcomes. The common support option was employed to limit testing of the balancing property to only treated mothers whose propensity scores for health facility delivery and EPNC were within the propensity score range. The average treatment effect on the treated (ATT) was estimated using the psmatch2 or teffects psmatch Stata command. To generate ATT, one-to-one nearest neighbor matching with replacement within a caliper range of ±0.01 was performed. Balancing tests were performed using the pstest stata command. For each covariate included in the propensity score estimation model, percentage (%) bias and % reduction bias were reported. Finally, a sensitivity analysis was undertaken to assess the assumption of the unconfoundedness of PSM using a Mantel–Haenszel bounds procedure.

## Results

### Background characteristics of study participants

A total of 90,830 women were included in the analysis. Of the total, 47.05% were aged 25–34 years and 43.27% had attained secondary education. In total, 95.43% of women were married and only 15.54% of them were head of the household. Almost half (49.07%) of the participants were from low-wealth quintile households (poorest and poorer). In total, 85.98% of women had a wanted pregnancy. In total, 58.72% of respondents watch television. Almost a third-fourth (73.08%) of participants were rural residents, and healthcare access was a big problem for more than half (51.45%) of them (Table 2).

**Table 2:** Socio-demographic characteristics of study subjects in LMICs.

**Variables**	**Categories**	**Antenatal care visits**	
		<**8 visits (%)**	≥**8 visits (%)**
Maternal age	15–24	32,662 (85.78)	5,413 (14.22)
	25–34	35,033 (81.97)	7,705 (18.03)
	35–49	8,490 (84.76)	1,527 (15.24)
Women's education	Not educated	22,398 (93.15)	1,646 (6.85)
	Primary	14,332 (90.63)	1,481 (9.37)
	Secondary	31,741 (80.76)	7,560 (19.24)
	Higher	7,714 (83.88)	3,958 (16.12)
Wealth status	Poorest	21,528 (90.90)	2,155 (9.10)
	Poorer	18,245 (87.36)	2,639 (12.64)
	Middle	15,101 (82.80)	3,136 (17.20)
	Richer	12,289 (78.42)	3,381 (21.58)
	Richest	9,022 (73.02)	3,334 (26.98)
Marital status	Not in a union	3,722 (89.62)	431 (10.38)
	Married	72,463 (83.60)	14,214 (16.40)
Pregnancy wanted	No	11,214 (88.06)	1,520 (11.94)
	Yes	64,971 (83.19)	13,125 (16.81)
Frequency of watching television	No	34,451 (91.87)	3,047 (8.13)
	Yes	41,734 (78.25)	11,598 (21.75)
Frequency of listening to a radio	No	55,143 (84.58)	10,050 (15.42)
	Yes	21,042 (82.08)	4,595 (17.92)
Frequency of reading newspaper/magazine	No	61,619 (86.47)	9,645 (13.53)
	Yes	14,566 (74.45)	5,000 (25.55)
Birth order	1	23,599 (80.10)	5,864 (19.90)
	2–5	44,787 (84.64)	8,128 (15.36)
	>6	7,799 (92.27)	653 (7.73)
Sex of household head	Male	64,163 (83.64)	12,553 (16.36)
	Female	12,022 (85.18)	2,092 (14.82)
Residence	Urban	18,276 (74.74)	6,178 (25.26)
	Rural	57,909 (87.24)	8,467 (12.76)
Accessing healthcare	Big problem	35,239 (79.92)	8,853 (20.08)
	Not big problem	40,943 (87.61)	5,791 (12.39)

### Estimated propensity scores and model significance

The mean propensity score was 0.16, with a standard deviation of 0.10 indicating the variability is little between the treatment and control groups. Regarding model significance, the pseudo-R2 before matching and after matching was 0.089 and < 0.001, respectively. Very low pseudo-R2 (< 0.001) indicates the distribution of covariates between both groups was similar (Table 3).

**Table 3:** Model significance and estimated propensity scores.

**Model significance**	**Sample**	**Pseudo-R2**	**LR chi2**	***p* > chi2**
	Unmatched	0.089	7180.52	0.000
	Matched	< 0.001	20.19	0.043
**Mean propensity score**		0.16		
**Standard deviation**		0.10		
**Region of common support**		0.015–0.565		

### Common support

**Figure 2:**
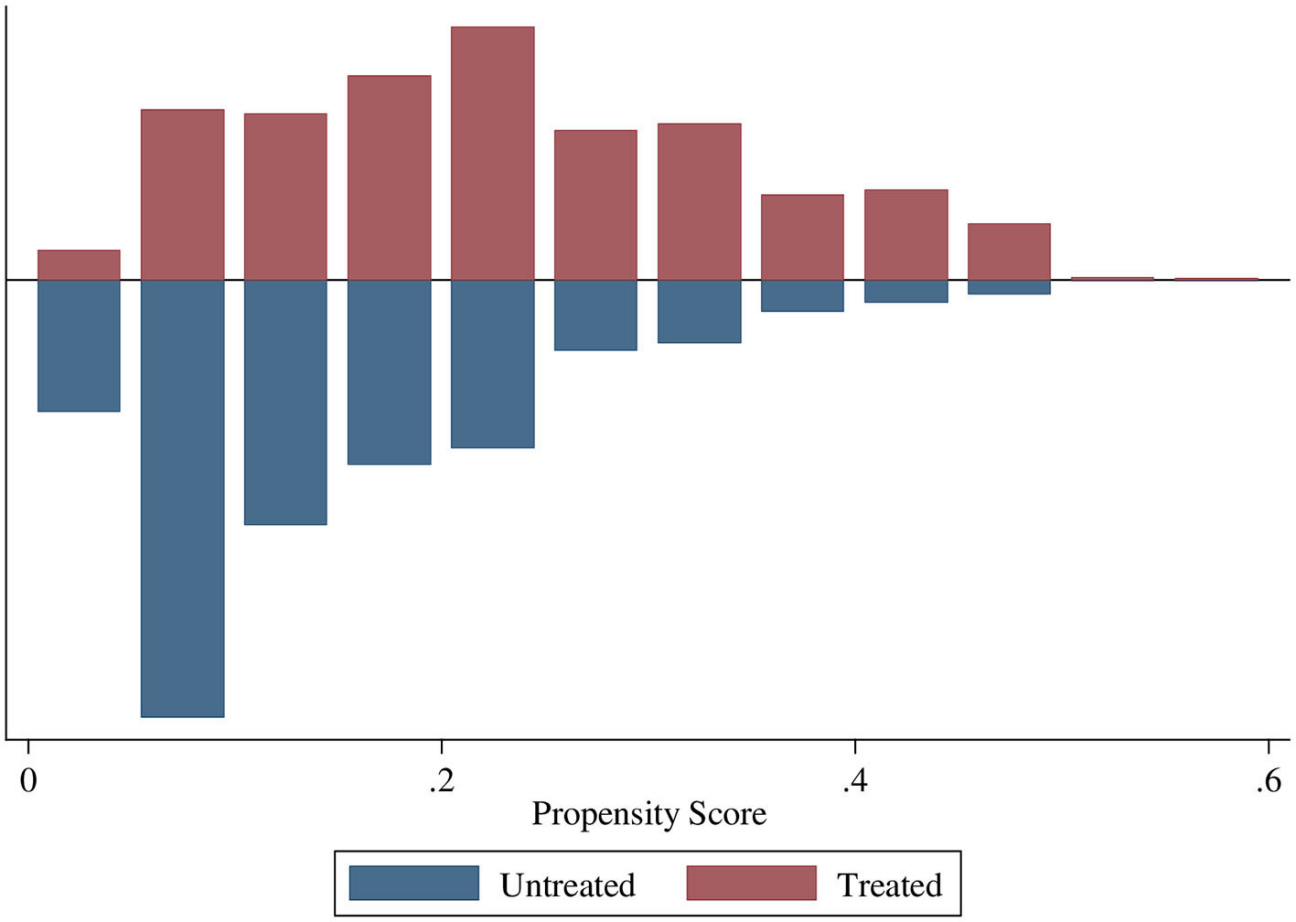
Propensity scores of ANC8+ by health facility delivery.

**Figure 3:**
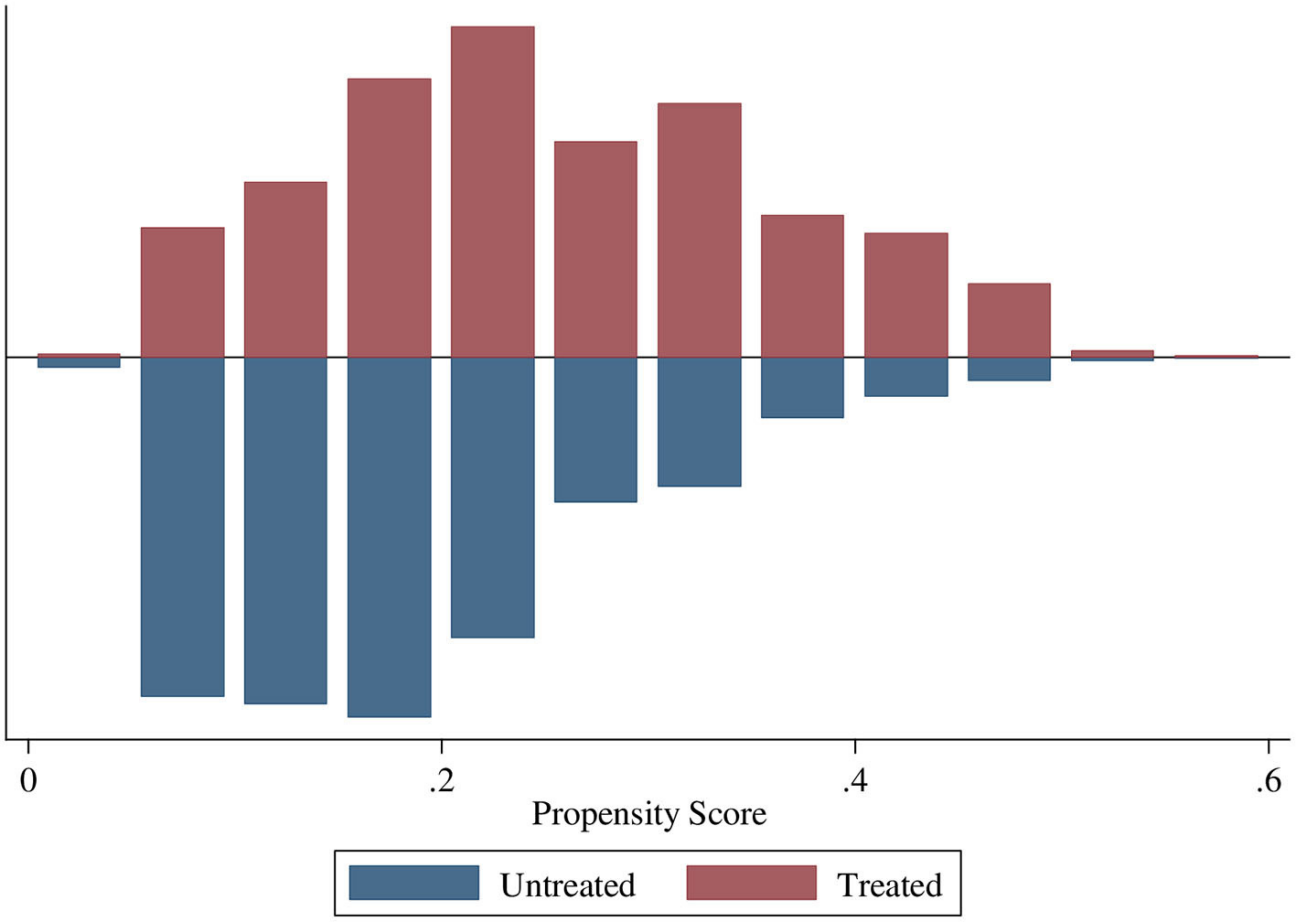
Propensity scores of ANC8+ by early PNC.

The region of common support ranges from 0.015 to 0.565. The common support improves the quality of matching by discarding individuals when there is no availability of a match. The treatment (ANC8 + visits) and control (<8 ANC visits) groups are matched and comparable as the quality of matching by health facility delivery and early PNC were balanced with no off support (Figures 2, 3).

### Average treatment effect of ANC8+ on health facility delivery and EPNC

Our study showed that the average treatment effect on the treated (ATT) on facility delivery and early PNC was 0.14 and 0.10, respectively. This indicates that the probability of facility-based delivery was 14% higher among treated women (who have ANC8+ visits) compared with the control group. Meanwhile, women who had ANC 8+ have an increased chance of having early PNC by 10% compared with those who had not. The net difference of ANC4 between treated and control was higher on institutional delivery; however, the ANC8+ had more impact on the EPNC. Generally, the ATT of ANC8+ has more impact in the treated (94%), while in ANC4+ it is 86% (Table 4).

**Table 4:** Estimation of average treatment effect on the treated, average treatment effect on the untreated, and average treatment effect of 8+ ANCs on facility delivery and early PNC.

**Variable**	**Sample**	**Treated**	**Control**	**Difference**	**SE**
ANC8+ on institutional delivery	Unmatched	0.94	0.76	0.18	0.004
	ATT	0.94	0.80	0.14	0.013
	ATU	0.76	0.88	0.11	
	ATE			0.12	
ANC8+ on early PNC	Unmatched	0.76	0.65	0.11	0.004
	ATT	0.72	0.62	0.10	0.016
	ATU	0.73	0.65	0.08	
	ATE			0.08	
ANC4+ on institutional delivery	Unmatched	0.86	0.68	0.18	0.002
	ATT	0.86	0.62	0.24	0.017
	ATU	0.68	0.82	0.14	
	ATE			0.19	
ANC4+ on early PNC	Unmatched	0.71	0.65	0.06	0.004
	ATT	0.71	0.64	0.07	0.016
	ATU	0.77	0.66	0.05	
	ATE			0.06	

### Balancing test

This study depicted before and after matching mean values and bias of each covariate in both groups. There is a percentage bias reduction for all matching covariates presented along with the *t*-test. The significance level of the *t*-test was also calculated, and it showed that the majority of covariates were balanced (Table 5).

**Table 5:** Comparison of the standardized differences of baseline characteristics before and after matching.

**Variable**	**Sample**	**Treated**	**Control**	**Bias**	**% reduction bias**	***t***	***p* > *t***
Age	Unmatched	1.7357	1.6827	8.0		8.74	0.000
	Matched	1.7347	1.7296	0.8	90.3	0.68	0.495
Education status	Unmatched	1.9443	1.3252	64.8		69.36	0.000
	Matched	1.9443	1.9375	0.7	98.9	0.65	0.514
Wealth status	Unmatched	3.2117	2.5935	45.4		50.46	0.000
	Matched	3.2117	3.2432	−2.3	94.9	−1.98	0.048
Marital status	Unmatched	0.97057	0.95114	10.0		10.31	0.000
	Matched	0.97057	0.97521	−2.4	76.1	−2.45	0.014
Pregnancy wanted	Unmatched	0.89621	0.8528	13.1		13.87	0.000
	Matched	0.89621	0.90345	−2.2	83.3	−2.06	0.039
Frequency of watching television	Unmatched	1.3908	0.91195	55.8		59.62	0.000
	Matched	1.3908	1.396	−0.6	98.9	−0.55	0.585
Frequency of listening to radio	Unmatched	0.45401	0.41214	5.8		6.41	0.000
	Matched	0.45401	0.45005	0.5	90.5	0.46	0.642
Frequency of reading newspaper/magazine	Unmatched	0.47054	0.25184	34.2		41.43	0.000
	Matched	0.47054	0.46064	1.5	95.5	1.19	0.232
Birth order	Unmatched	1.6442	1.7926	−25.3		−27.39	0.000
	Matched	1.6442	1.6422	0.3	98.7	0.30	0.763
Sex of household head	Unmatched	1.1428	1.1578	−4.2		−4.58	0.000
	Matched	1.1428	1.1393	1.0	76.3	0.87	0.383
Residence	Unmatched	1.5781	1.7601	−39.4		−46.00	0.000
	Matched	1.5781	1.5802	−0.4	98.9	−0.36	0.723
Accessing healthcare	Unmatched	0.55401	0.51214	5.8		6.41	0.000
	Matched	0.55401	0.55005	0.5	90.5	0.46	0.642

### Sensitivity analysis for hidden bias

The sensitivity analysis was performed using the Mantel–Haenszel statistic to estimate the extent of unobservable covariates biases on our inferences about the effects of 8+ ANC visits. The Q_mh+ statistic adjusts the MH statistic downward for positive unobserved selection bias, and as the estimated ANC visits effect is positively related to outcome variables, we focused on the positive bias. Based on this, a critical value >1.4 indicates that the result will be insensitive and the conclusion could be uncertain (Supplementary Table).

## Discussion

Maternal and child mortality remain a substantial public health concern worldwide, especially in LMICs (2, 32, 33). Complications during pregnancy and childbirth are the leading causes of maternal and child deaths (34, 35). The WHO recommended ANC8+ visits as a key strategy to endorse pregnant women's health (9). In addition to ANC visits, health facility delivery and early PNC have been reported as major contributors to preserving women's and child's health (28, 36, 37). Previous studies showed that prenatal care is an imperative factor for subsequent healthcare utilization (38–40). This study revealed that ANC8+ visits have a significant and positive influence on health facility delivery as well as EPNC.

We used an innovative statistical method called the propensity score matching (PSM) to understand the net impact of ANC8+ visits on health facility delivery and EPNC. This method is useful to ensure that study participants in the control and treatment groups are similar based on measured characteristics. PSM reduces selection bias and offers an alternative for measuring treatment effects in cross-sectional/observational/non-experimental data when randomized clinical trials are not possible or unethical. Therefore, the groups are the same except for the treatment variable ensuring a less-biased estimate of treatment effects.

This study found that women who attended ANC8+ visits had a 14% higher chance of having their delivery at health facilities. Our finding was supported by previous studies conducted in the developing world (41–43). For example, a study from Uganda assessed the impact of ANC visits based on previous WHO recommendations (ANC4+ visits), and the result showed that the treated women were almost 12 and 10% higher to give birth at health facilities and have EPNC, respectively (26). This is likely due to the fact that women who attend adequate ANC receive maternal education and are often referred to health facilities for delivery. However, this result showed that ANC4+ contact has more impact (24%) on health facility delivery. Generally, we can conclude that adequate ANC contacts are critical for institutional delivery. Therefore, this health facility delivery in turn could advantageous for pregnant women to attend early PNC.

This study further showed that women who had ANC8+ visits were associated with a 10% higher probability of early PNC compared with their counterparts. ANC8+ contact showed a strong impact (10%) compared with ANC4+ contacts (7%) on EPNC. In LMICs, PNC is one of the most underutilized and weakest of all maternal and child health services. The result aligns with previous studies, which highlighted a positive association between ANC8+ visits and early PNC utilization (26, 44). This is probably because frequent contact with healthcare providers throughout the ANC period would create an understanding of the importance of attending early PNC.

Our study has strengths and weaknesses. Among the strengths, we used a large sample and conducted a propensity score matching to reduce selection bias between groups based on observable confounders. This gives better and unbiased estimates of the treatment effects of ANC8+ visits. Even though including women who had given birth within the year prior to the surveys is the strength of this study to reduce recall bias compared with previous studies that included women who had given birth within 5 years prior to the surveys, still recall bias could happen (ANC8+). Among the weaknesses, although PSM removes bias based on observable variables, bias due to unobservable confounders (hidden bias) is not accounted for and could lead to overestimated effects of the treatment on outcome variables. Moreover, important covariates which may directly affect the net impact such as women with a high risk of pregnancy were not included.

## Conclusion and recommendation

Our study confirmed that ANC8+ visits significantly increased the likelihood of health facility-based delivery and early PNC utilization in LMICs. Health policymakers and healthcare providers should focus on and target pregnant women to attend adequate ANC visits (according to revised WHO recommendation) as our study indicates that ANC8+ visits significantly improved the chances of subsequent care, particularly the early PNC compared with the ANC4 visits.

## Data availability statement

Publicly available datasets were analyzed in this study. This data can be found here: https://dhsprogram.com/data/dataset_admin/index.cfm.

## Author contributions

DC: conceptualization. DC, FA, DB, MA, MM, AK, NT, and AA: study design, execution, acquisition of the data, analysis and interpretation, writing, and review and editing. All authors contributed to the article and approved the submitted version.
