# Supplementary material for: Effectiveness of eight or more antenatal contacts on health facility delivery and early postnatal care in low- and middle-income countries: a propensity score matching
Source: Front Med (Lausanne). 2023 Jul 21;10:1107008. doi: 10.3389/fmed.2023.1107008 (PMC10401271; doi:10.3389/fmed.2023.1107008)
Supplement: Supplementary file 1 [file Table_1.pdf]

Supplementary table. Sensitivity analysis using Mantel-Haenszel

Gamma (Γ)	Q_mh+	Q_mh-	P_mh+	P_mh-
1	3.119	3.119	0.001	0.001
1.1	2.697	3.548	0.003	0.000
1.2	2.317	3.946	0.010	0.000
1.3	1.971	4.319	0.024	0.000
1.4	1.654	4.670	0.049	0.000
1.5	1.360	5.003	0.087	0.000
1.6	1.087	5.318	0.138	0.000
1.7	0.832	5.621	0.203	0.000
1.8	0.591	5.909	0.277	0.000
1.9	0.365	6.187	0.357	0.000
2	0.149	6.455	0.441	0.000

Gamma : odds of differential assignment due to unobserved factors

Q_mh+ : Mantel-Haenszel statistic (assumption: overestimation of treatment effect)

Q_mh- : Mantel-Haenszel statistic (assumption: underestimation of treatment effect)

p_mh+ : significance level (assumption: overestimation of treatment effect)

p_mh- : significance level (assumption: underestimation of treatment effect)
